# Regular Practice of Autogenic Training Reduces Migraine Frequency and Is Associated With Brain Activity Changes in Response to Fearful Visual Stimuli

**DOI:** 10.3389/fnbeh.2021.780081

**Published:** 2022-01-21

**Authors:** Dóra Dobos, Edina Szabó, Dániel Baksa, Kinga Gecse, Natália Kocsel, Dorottya Pap, Terézia Zsombók, Lajos R. Kozák, Gyöngyi Kökönyei, Gabriella Juhász

**Affiliations:** ^1^SE-NAP 2 Genetic Brain Imaging Migraine Research Group, Hungarian Brain Research Program, Semmelweis University, Budapest, Hungary; ^2^Department of Pharmacodynamics, Faculty of Pharmacy, Semmelweis University, Budapest, Hungary; ^3^Center for Pain and the Brain (PAIN Research Group), Department of Anesthesiology, Critical Care and Pain Medicine, Boston Children’s Hospital and Harvard Medical School, Boston, MA, United States; ^4^Institute of Psychology, ELTE Eötvös Loránd University, Budapest, Hungary; ^5^Magnetic Resonance Research Center, Semmelweis University, Budapest, Hungary; ^6^NAP-2-SE New Antidepressant Target Research Group, Hungarian Brain Research Program, Semmelweis University, Budapest, Hungary; ^7^MTA-SE Neuropsychopharmacology and Neurochemistry Research Group, Hungarian Academy of Sciences, Semmelweis University, Budapest, Hungary

**Keywords:** migraine, autogenic training, emotional processing, stress, fMRI

## Abstract

Several factors can contribute to the development and chronification of migraines, including stress, which is undoubtedly a major trigger. Beyond pharmacotherapy, other treatment methods also exist, including behavioral techniques aiming at reducing patients’ stress response. However, the exact brain mechanisms underlying the efficacy of such methods are poorly understood. Our pilot study examined whether the regular practice of autogenic training (AT) induces functional brain changes and if so, how it could be associated with the improvement of migraine parameters. By exploring neural changes through which AT exerts its effect, we can get closer to the pathomechanism of migraine. In particular, we investigated the effect of a headache-specific AT on brain activation using an implicit face emotion processing functional MRI (fMRI) task in female subjects with and without episodic migraine. Our focus was on migraine- and psychological stress-related brain regions. After a 16-week training course, migraineurs showed decreased activation in the migraine-associated dorsal pons to fearful compared with neutral visual stimuli. We also detected decreasing differences in supplementary motor area (SMA) activation to fearful stimuli, and in posterior insula activation to happy stimuli between healthy subjects and migraineurs. Furthermore, migraineurs reported significantly less migraine attacks. These brain activation changes suggest that AT may influence the activity of brain regions responsible for emotion perception, emotional and motor response integration, as well as cognitive control, while also being able to diminish the activation of regions that have an active role in migraine attacks. Improvements induced by the training and the underlying neurophysiological mechanisms are additional arguments in favor of evidence-based personalized behavioral therapies.

## Introduction

Being the third most prevalent disorder in the world, migraines affect more than 10% of the population (Bohm et al., 2018). In the case of migraines, a throbbing or pulsating headache can be accompanied by nausea, vomiting, sensitivity to light, sound, and odors. The condition is a cause of disability to its sufferers since an attack can last from several hours to several days, hampering everyday activity. There are sex differences in the prevalence of migraines, with women being affected more than men worldwide (Woldeamanuel and Cowan, 2017), nonetheless, the exact causes of migraines are still unknown.

The pharmacotherapy of migraines consists of an acute treatment aimed at relieving pain and preventive therapy (prophylaxis) that intends to reduce the frequency and intensity of attacks. A potential complication of frequent acute pharmacotherapy is medication overuse headache induced by the regular intake of high doses of non-specific or specific anti-migraine drugs (Goadsby and Sprenger, 2010). Moreover, migraine-specific triptans may have, e.g., cardiovascular side effects (Goadsby and Sprenger, 2010), which can be a reason for contraindication or discontinuance of treatment (Wells et al., 2014). In recent years, a series of promising new anti-migraine targets has appeared (e.g., calcitonin gene-related peptide (CGRP) receptor antagonists, anti-CGRP monoclonal antibodies, anti-PACAP antibodies; Do et al., 2019), and some of them can be used in prophylaxis. Despite these developments, only a negligible proportion of patients receives appropriate preventive therapy at the moment (Katsarava et al., 2018). This can be explained by several factors including poor adherence of patients when taking prophylactics due to side effects and lack of efficacy (Blumenfeld et al., 2013). However, the application of effective preventive therapies is a matter of primary importance to avoid the chronification of headaches, increase of pain intensity, and not least to cut the economic costs of migraines (Yu et al., 2010). Therefore, evidence-based behavioral therapies could be viable alternatives or supplements to drug therapies (Holroyd et al., 2010).

Behavioral interventions may increase the adherence to pharmacotherapies in a safe and well-tolerated manner (Minen et al., 2018). Zsombok et al. (2003) proved that a combination of evidence-based methods [autogenic training (AT), elements of cognitive therapy, and imaginative training] customized for patients with headaches can improve the management of the disease. As a result of their study, both the headache frequency and medication consumption of patients with headaches significantly decreased by the end of a 4-month-long training period (Zsombok et al., 2003). Schultz-type AT (Linden, 1993) is one of the oldest behavioral techniques helping patients manage stress, a common trigger of migraine (Borsook et al., 2012). It is an autosuggestive relaxation method during which patients learn to take control of their stress response or at least perceive stress triggers and try to react adaptively (Linden, 1993) (For a detailed description of the applied training, see Zsombok et al., 2003 or section “Autogenic Training Developed for Headache Patients,” and Supplementary Methods). A way of measuring the efficacy of AT is the investigation of activation changes in brain regions involved in stress processing.

The stress response is mediated by the neuroendocrine system, the hypothalamic-pituitary-adrenal (HPA) axis that is regulated by a negative feedback mechanism, and also by a network of brain areas. The most important elements of this network are the medial prefrontal cortex, the hippocampus, and the amygdala (Dedovic et al., 2009) which are also involved in nociceptive and emotional processing (Garrett and Maddock, 2006; Onozawa et al., 2011). Imaging studies reported altered brain activity in migraineurs as compared with healthy subjects in experimental paradigms where psychological stress was represented by negative emotional stimuli. Wilcox et al. (2016) found increased activation in the hippocampus, amygdala, frontal medial cortex, middle frontal gyrus, and cingulate gyrus to aversive emotional stimuli. Higher activation in the middle frontal gyrus to fearful stimuli was also detected by Szabó et al. (2019). These observations may suggest an altered processing of aversive stimuli in migraineurs. There is some evidence in the literature regarding the effects of behavioral therapies on the activity of these areas, but prior research has focused solely on real-time neural changes within healthy subjects during training practice. For example, Kobayashi and Koitabashi (2016) found decreased neural activity in the cingulate cortex of healthy males during progressive muscle relaxation (PMR) (a technique similar to AT) compared with a control session, demonstrating that practicing PMR in the scanner can change brain activation. In another study, a group of subjects with experience in AT in comparison to subjects without experience showed increased prefrontal cortical activation during AT, suggesting improved top-down cortical control of perception or altered emotional processing (Schlamann et al., 2010). Considering these results, we hypothesized that an altered activation of brain areas associated with psychosocial stress might be achievable in the case of both a control group and a migraine group following training in and regular practice of the headache-specific AT method described in Zsombok et al. (2003). In addition, while investigating reactions to a painful stimulus, Naglatzki et al. (2012) showed that the AT session silenced activations in regions involved in pain processing (anterior midcingulate cortex, right thalamus, anterior insular cortices, and left caudate nucleus). Thus, we also expected that AT might influence the activity of brain regions contributing to the modulation of pain during headaches.

For a long time, the migraine generation has primarily been attributed to the hypothalamus and pons. Meanwhile, the altered activation of these regions has been demonstrated in other migraine stages too. Increased activation of the hypothalamus has been found in the pre-ictal stage (preceding attacks), which might explain why certain hypothalamus-driven homeostatic functions like sleep or feeding can trigger migraine attacks, while the pons has been proved to be active even during the headache phase (Maniyar et al., 2014; Schulte and May, 2016). In contrast, Denuelle et al. (2007) showed that both the hypothalamus and dorsomedial pons remain active after the alleviation of pain with sumatriptan. Wilcox et al. (2016) measured an increased pontine response to negative emotional stimuli in migraineurs compared with control subjects during the interictal (pain-free) period. As a result of the AT, we expected a decrease in the activation of brain regions active before or during migraine attacks in migraine patients, as well as a reduction in the number of migraine attacks.

Emotional processing is typically investigated by presenting the subjects with pictures of human faces expressing feelings like happiness, sadness, anger, and fear (Andreatta et al., 2012; Szabó et al., 2019). In our pilot study, we used an implicit emotional processing functional MRI (fMRI) task, and we investigated whether the regular practice of AT has any effect on brain activation patterns. Specifically, we focused on brain areas associated with aversive emotional processing, emotional stress processing, and pain generation, as well as the above-mentioned AT-related areas.

## Materials and Methods

### Participants

A total of 26 healthy controls and 15 episodic migraine participants recruited through headache clinics and advertisements were included in the study. All of the subjects were right-handed (assessed by the Edinburgh Handedness Inventory; Oldfield, 1971), aged between 20 and 37 years, and free of neurological, psychological [checked using the Mini-International Neuropsychiatric Interview (M.I.N.I.); Sheehan et al., 1998], and chronic diseases (except migraines). Only episodic migraineurs without aura were selected. The International Classification of Headache Disorders-III criteria (Headache Classification Committee of the International Headache Society [IHS], 2013) were used to check for this criterion. In addition, all the inclusion criteria were tested for by psychologists and headache specialists. Following the first round of fMRI scanning, participants learned AT developed for patients with headaches, and the second round of scanning was carried out 4 months later. Subjects took no analgesics 48 h before the scan, nor prophylactic medicines from the last 3 months prior to the study until the end. Patients with migraine were attack-free 48 h prior to, and 24 h after the scan. Two participants were excluded due to movement artifacts, and a further three due to other reasons such as a panic attack or analgesic consumption before the scan. One subject did not practice AT regularly, therefore, she also had to be omitted. Since the majority of migraineurs were female (1 male out of 12 patients), we excluded all male participants from the study. The final sample thus consisted of 15 female controls and 11 female migraineurs. Note that since the age of certain subjects was different at the time of the first and the second round of fMRI scanning, the ages of test subjects during the latter round were considered for mean age calculation and fMRI-based statistical analyses.

The study protocol was approved by the Scientific and Research Ethics Committee of the Medical Research Council of Hungary (23609-1/2011-EKU; 23421-1/2015/EKU), and written informed consent forms were obtained from all subjects in accordance with the Declaration of Helsinki.

### Autogenic Training Developed for Headache Patients

Controls and migraineurs without history of preventive headache therapy participated in a 16-week-long AT course combined with elements of cognitive therapy and imaginative training. The control subjects were offered to learn this stress-relieving technique, while migraineurs received it as part of their treatment. Both groups participated on a voluntary basis. The inclusion of cognitive therapy elements was supported by previous studies demonstrating the effectiveness of combined therapies among patients with headaches (Attanasio et al., 1987). By including such elements, we aimed to identify and modify maladaptive cognitive coping responses.

The 50-min training sessions were conducted by a headache-specialist neurologist-psychiatrist having extensive psychotherapy experience (T. Z.) and were repeated on a weekly basis (see also Supplementary Methods). Classical Schultz-type AT (Linden, 1993) was taught to the participants in 12 sessions supplemented by a cognitive coping skills training focusing on coping self-statements, cognitive reappraisal, and pleasant imagery. Subjects were encouraged to share their personal experiences and/or problems. Additional sessions were dedicated to special imaginative training that aimed at relaxing and heating areas of the back, shoulders, and neck. The therapist demonstrated the three layers of back muscles, then relaxation and heating were applied to each layer, one by one. The 14th session concentrated on the head and was followed by two confirmatory sessions. Since neck pain is a frequent symptom of migraineurs (Florencio et al., 2014), and muscle stiffness is also associated with migraines (Tali et al., 2014), focusing on the head, neck, and shoulders could be particularly beneficial for patients. During the 15th session, the experience of happiness and during the 16th session, an imaginative exercise of shrinking and enlargement of the body were additionally introduced to increase body awareness (see Zsombok et al., 2003).

During each encounter, the therapist checked the participants’ compliance verbally, because only those who completed all sessions and practiced at least once a week during 4 months were eligible for the study.

### Clinical Measures

The migraine participants were requested to characterize their headache by the following features: age at migraine onset, number of years with migraines, migraine frequency (average number of migraine attacks per month), and migraine laterality. The number of attacks in the month prior to both rounds of fMRI scanning was also recorded.

### Functional MRI Task Design

In an 8-min session, gray-scale photographs of human faces were shown to the participants who categorized the sex of faces expressing four types of emotion: fear, happiness, sadness, and neutrality. For this implicit emotional processing task, the photographs were selected from the standard Ekman photographs of emotions (Ekman and Friesen, 1976), all of them having a black monochrome background, and body parts such as the hair and ears were hidden. The experimental paradigm consisted of 21 emotional blocks: fearful, happy, and sad photographs presented in a pseudo-random order and scattered by neutral blocks. The blocks were 20-s-long and presented six faces (three males and three females) for 3,000 ms each with an interstimulus interval of 333 and 334 ms. Four different people’s faces were combined in each block. Three 20-s-long rest blocks separated the emotional photographs while a white fixation cross was displayed at the center of the dark screen. The participants were familiarized with the task prior to the scans, using neutral faces on a laptop. Inside the magnetic resonance imaging (MRI) machine, subjects used a two-button device to indicate the sex of the faces (Szabó et al., 2019).

The presentation of the stimuli and data collection (accuracy and response time) were performed using the *E*-Prime 2.0 software (Psychology Software Tools, Inc., Pittsburgh, PA, United States).

Each participant performed the task twice: before the AT course and after the course.

### Functional MRI Data Acquisition

Imaging was conducted on a 3T MRI scanner (Achieva 3T, Philips Medical System, Philips Healthcare, Amsterdam, Netherlands) using a BOLD-sensitive T2*-weighted echo-planar imaging sequence (TR = 2,500 ms, TE = 30 ms, and FOV: 240 mm × 240 mm) with 3 mm × 3 mm in-plane resolution and contiguous 3-mm slices providing whole-brain coverage. A series of anatomical images were also acquired during the functional imaging session using a T1-weighted 3D TFE sequence with 1 mm × 1 mm × 1 mm resolution.

### Region of Interest Definition

Being particularly interested in the potential impact of AT on migraine-related brain areas, we examined the neural response to emotional faces within a region of interest (ROI) in the migraine subsample. Regions active before or during migraine attacks, referred to as *migraine-related regions*, were used as a mask during the data analysis. The ROI consisted of the hypothalamus and the pons, two brain areas that can have a decisive role in the onset and sustainment of migraine pain, showing altered functioning both in painful and pain-free stages (Denuelle et al., 2007; Maniyar et al., 2014; Schulte and May, 2016; Wilcox et al., 2016). It was generated using the toolboxes of Statistical Parametric Mapping software package (SPM12, The Wellcome Centre for Human Neuroimaging, UCL Queen Square Institute of Neurology, London, United Kingdom^1^): WFU_PickAtlas v. 3.0.5 (The Functional MRI Laboratory, Wake Forest University School of Medicine, NC, United States) and MarsBaR v. 0.44 (Brett et al., 2002).

### Demographic and Clinical Data Analysis

IBM SPSS Statistics version 25 (SPSS, Inc., Chicago, IL, United States) was used to analyze the demographic and clinical data of participants. See Table 1 for details.

**TABLE 1:** Demographic and clinical characteristics of migraineurs and healthy controls.

	Migraineurs (*n* = 11)		Controls (*n* = 15)		Test statistics	*P*-value
	Mean (SD)	Range	Mean (SD)	Range		
**Age at second fMRI scan**	26.91 (3.53)	22–33	24.80 (2.91)	22–30	1.669	0.108
**Highest education**					0.158	0.691
High school	5 (45%)		8 (53%)			
Graduate degree	6 (55%)		7 (47%)			
**Migraine laterality**						
Unilateral	6 (55%)					
Bilateral	5 (45%)					
**Age at migraine onset**	16.14 (7.13)	3–26				
**Number of years with migraine at second fMRI scan**	10.77 (6.36)	3–23				
**Migraine attacks per month**	4.77 (4.27)	1–12				
**Number of attacks in the month prior to the**						
First fMRI scanning	6.14 (3.02)	3–11				
Second fMRI scanning	3.45 (2.46)	0–8				
**Difference**					**2.679**	**0.007**
**AT frequency per week**	4.64 (3.67)	1–14	4.40 (3.44)	1–14	91.5	0.646

*SD, standard deviation. The P-values are based on independent samples t-test for age at second fMRI scan, Pearson’s Chi-squared test for highest education, Wilcoxon signed-rank test for the number of attacks, and Mann-Whitney U-test for AT frequency. Bold values represent the significant difference between the number of attacks in the month prior to the first and second fMRI scanning.*

### Functional MRI Data Analysis

Both preprocessing and statistical analysis of fMRI data were carried out in SPM12. After data conversion to NifTI format, the following steps of preprocessing were performed: realignment, co-registration of T1-weighted anatomical images to the mean of T2*-weighted functional ones, segmentation into different tissue types, spatial normalization to standard stereotaxic [Montreal Neurological Institute (MNI)] space, and spatial smoothing using an 8 mm full width at half maximum (FWHM) Gaussian kernel.

The motion outliers were detected using Artifact Detection Tools (ART^2^). Time points of individual subjects were considered as outliers if the global signal lied more than 3 standard deviations away from its mean or the scan-to-scan movement was greater than 1 mm. If the proportion of the total outliers exceeded 15%, the subject’s data were excluded from further investigation. Two subjects were excluded due to motion artifact. During the first-level statistical analysis, both motion parameters and outliers were treated as covariates of no interest and were regressed out.

The statistical analysis was performed using the General Linear Model for three types of contrast: (1) fearful-neutral, (2) happy-neutral, and (3) sad-neutral. Since the participants were scanned twice, before starting and after completing AT, the outcome was a before AT and an after AT series of contrast images. Contrast maps used for the between-groups and within-group analyses resulted from the subtraction of the before AT contrast images from the after AT contrast images. The subtraction of individually paired images was carried out in Matlab version 2016b (MathWorks, Natick, MA, United States).

Between-group analyses were performed using a full factorial statistical design to shed light on differences in activation change between the migraineur and the control group. Within-group analysis was carried out with a one-sample *t*-test in *migraine-related regions* in the migraine subsample. All statistical tests were run for all the above-mentioned contrasts, and the age of the participants was controlled as a covariate to exclude its potential impact on the alteration patterns (Vetvik and MacGregor, 2017). Initially, a height threshold of uncorrected *p* < 0.001 was applied in every analysis, but only results controlled for family wise error rate (*p*_FWE_ < 0.05) were considered as statistically significant (Bennett et al., 2009). Cluster size was adjusted to the extent of the ROI: 10 voxels for the between-group analyses and 5 voxels for *migraine-related regions*, since brainstem nuclei are hardly distinguishable due to their small size (Beissner, 2015). However, the restriction of the analyses to even smaller areas would have risked missing activations or deactivations. Inferences were exclusively made based on voxels surviving correction for multiple comparisons on the cluster-level in between-group analyses and on peak-level within *migraine-related regions*. Significant clusters were identified in WFU_PickAtlas v. 3.0.5 (The Functional MRI Laboratory, Wake Forest University School of Medicine, NC, United States) using the Automated Anatomical Labeling atlas (aal; Tzourio-Mazoyer et al., 2002) and displayed in MRIcroGL^3^ on the MNI152 template (Rorden and Brett, 2000). The beta values of significant voxels in *migraine-related regions* were extracted and correlated with the change in the number of migraine attacks and also with the number of migraine headaches in the month prior to the first round of fMRI scanning using SPSS.

## Results

### Clinical Evaluation

There was no difference in either age or AT frequency between the controls and migraineurs. The number of migraine attacks in the month prior to the second round of fMRI scanning was significantly lower than prior to AT (see Supplementary Table 1 for intra-individual changes). None of the healthy controls had migraine attacks after AT. The demographic and clinical characteristics of the migraine and control groups are detailed in Table 1. Among the migraineurs with unilateral headache, two subjects have left-sided, one subject has right-sided migraine, and the headache side of three subjects changes from attack to attack.

The mean accuracy level of the sex identification fMRI task was high both before AT and after AT. No significant differences were observed between migraineurs and healthy controls in mean accuracy and reaction times. Similarly, within-group analyses did not reveal any significant difference comparing the before AT and after AT mean accuracy level and reaction times. For details, see Supplementary Table 2.

### Emotional Faces Task-Related Activation

The main effects of the fMRI task are reported in the Supplementary Table 3. Activated areas in our study correspond well with previous findings (Fusar-Poli et al., 2009; Szabó et al., 2019).

### Between-Groups Analysis

**FIGURE 1:**
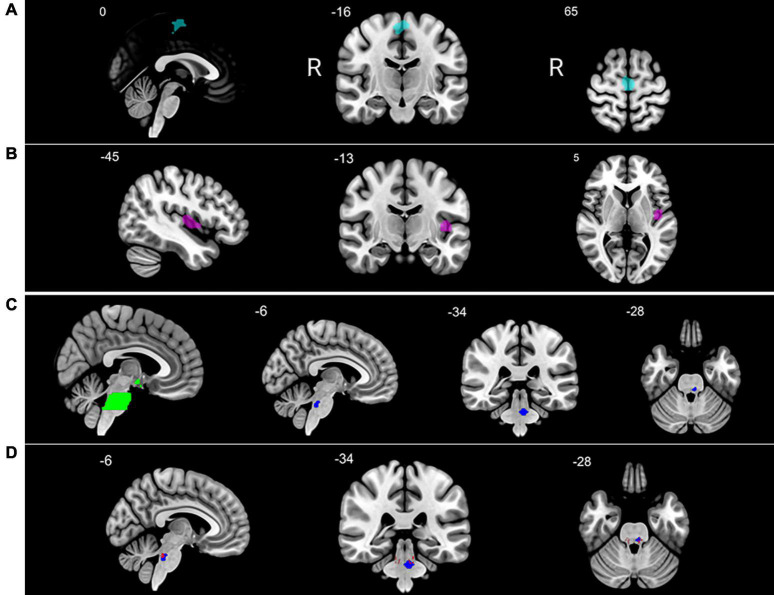
Brain regions showing altered activation after autogenic training (AT) course completion. After AT vs. before AT: **(A)** increased activation of left medial frontal gyrus [supplementary motor area (SMA)] in the control group to fearful emotional stimuli as compared with migraine patients; **(B)** increased activation of the left insula in migraineurs to happy emotional stimuli as compared with healthy controls; **(C)** region of interest (ROI) analysis in migraine-related regions in the migraine subsample: pons and hypothalamus ROI (green), decreased activation in the left pons of migraineurs to fearful emotional stimuli (blue); **(D)** left pons (blue) overlapping with parabrachial complex (red). Significant clusters are shown at *p*_FWE_ < 0.05 in Montreal Neurological Institute (MNI) coordinates (R = right side of the brain).

**FIGURE 2:**
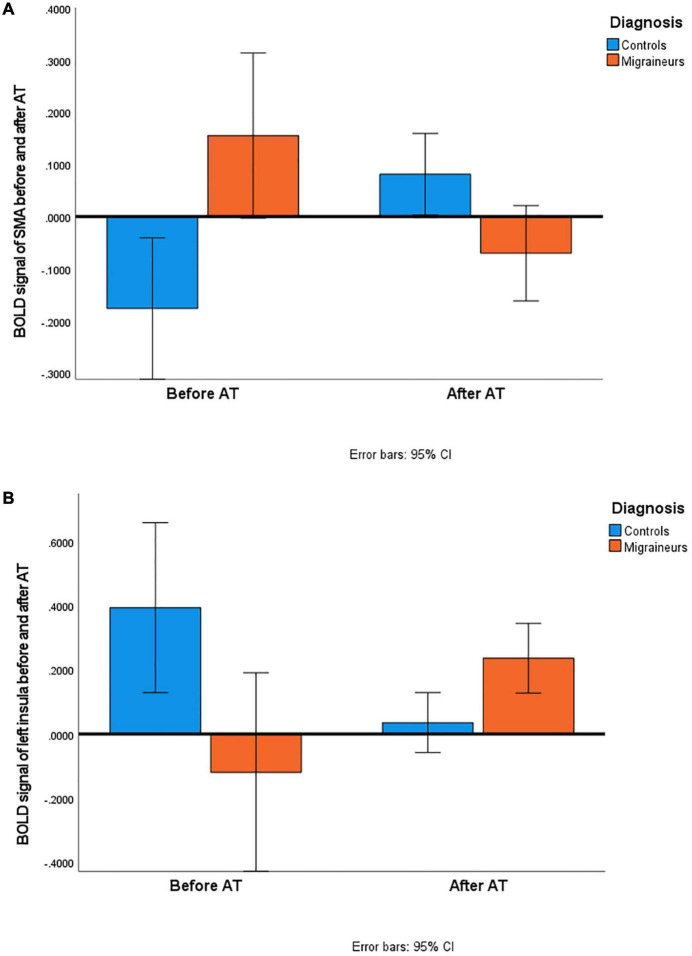
Comparison of BOLD signals in controls and migraineurs before and after AT. **(A)** Activation of left medial frontal gyrus [supplementary motor area (SMA)] increased in controls and decreased in migraineurs in response to fearful faces by the end of the 16-week AT. **(B)** Activation of left insula increased in migraineurs and decreased in controls in response to happy faces by the end of the 16-week AT. There is a significant difference between BOLD signal of controls and migraineurs both before AT (SMA: Mann-Whitney *U*-test = 23, *p* = 0.002; insula: Mann-Whitney *U*-test = 21, *p* = 0.001) and after AT (SMA: Mann-Whitney *U*-test = 42, *p* = 0.036; insula: Mann-Whitney *U*-test = 36, *p* = 0.016). The difference decreased by the end of the AT course in both cases.

Comparison of activation changes between groups revealed two significant differences. The activation of a region in the left medial frontal gyrus (see Figure 1A and Table 2) of controls significantly increased to fearful faces compared with migraineurs (cluster level, *p*_FWE_ < 0.05), while the activation of the left insula (see Figure 1B and Table 2) significantly increased to happy faces in migraineurs compared with controls (cluster level, *p*_FWE_ < 0.05). In a *post hoc* test, we compared the before AT and after AT activation levels of these areas in the control and migraine groups separately and found the different directions of activation change in the two groups in both cases (see Figures 2A,B). Intragroup comparisons showed significant changes within each area (see Supplementary Table 4), and the intergroup activation level difference decreased by the end of AT (see Figures 2A,B and Supplementary Table 5). Furthermore, we compared neutral faces to the rest condition to investigate significant neutral face processing differences between the two groups. Two-sample *t*-tests showed no significant differences between controls and migraineurs in neutral stimuli processing (*p*_FWE_ > 0.05) either before, or after AT. Therefore, it is unlikely that the two groups process neutral faces stimuli in a different manner, and this cannot be the source of findings in fearful and happy conditions. There was no correlation between AT frequency and either left medial frontal gyrus (Kendall’s tau b: 0.161, *p* = 0.284) or left insula (Kendall’s tau b: 0.174, *p* = 0.245) activation change.

**TABLE 2:** Brain areas showing different activation changes in healthy controls compared to migraineurs in response to fearful and happy emotional stimuli after autogenic training (AT) completion.

Cluster size	Cluster *p*_FWE_	Region	Peak coordinates			Peak *F*-value
			x	y	z	
**Controls > Migraineurs, increased activation, fearful faces**						
69	0.026	L medial frontal gyrus	0	−16	65	32.22
**Migraineurs > Controls, increased activation, happy faces**						
50	0.073	L Insula	−45	−13	5	20.38
*Positive interaction:*						*Peak T-value*
71	0.041	L Insula	−45	−13	5	4.51

*Significant clusters are shown at p_FWE_ < 0.05 in Montreal Neurological Institute (MNI) coordinates (L = left).*

Sad facial expressions did not reveal any difference between brain activation changes of control and migraine groups.

### Region of Interest Analysis in Migraine-Related Regions

Separately in migraineurs, the comparison of activation before starting and after completing AT course resulted in a decreased activation in the pons (see Figure 1C and Table 3) using the *migraine-related regions* mask (peak level, *p*_FWE_ < 0.05). According to the Harvard Ascending Arousal Network Atlas (Edlow et al., 2012), this pontine area overlaps with the parabrachial complex (see Figure 1D).

**TABLE 3:** Regions of interest (ROI) analysis in migraine-related regions: dorsal pons showing a significantly decreased activation in response to fearful emotional stimuli after autogenic training (AT) completion in migraineurs.

Cluster size	Peak *p*_FWE_	Region	Peak coordinates			Peak *T*-value
			x	y	z	
**(a) *Before controlling for the difference in number of attacks in the month prior to the first and second fMRI scanning***						
12	0.030	L Pons	−6	−34	−28	6.12
**(b) *After controlling for the difference in number of attacks in the month prior to the first and second fMRI scanning***						
12	0.007	L Pons	−6	−34	−28	8.12

*Significant clusters are shown at p_FWE_ < 0.05 in Montreal Neurological Institute (MNI) coordinates (L = left).*

*Post hoc* tests showed that no similar significant differences could be detected in the control group or testing happy-neutral or sad-neutral contrast in either group.

*Post hoc* analyses also demonstrated that the activation decrease remained significant after controlling for the difference in the number of attacks in the month prior to the first and second fMRI scanning (see Table 3), and there was no correlation between pons activation change and either difference in the number of attacks (Spearman’ rho: −0.419, *p* = 0.199) or AT frequency (Spearman’s rho: 0.354, *p* = 0.286).

There was a trend in positive correlation (Spearman’ rho: 0.541, *p* = 0.085) between the pons deactivation and the number of attacks in the month prior to the first fMRI scanning: those having a lower number of attacks showed a greater decrease in pons activation (see Supplementary Figure 1).

## Discussion

The cortical hyperexcitability of migraineurs is not limited to sensory stimuli, but it has also been observed in relation to emotional ones (Wilcox et al., 2016; Wang et al., 2017; Szabó et al., 2019). The increased sensitivity can limit adaptive coping with emotionally stressful situations and may also be associated with the pathophysiology of the disorder (Chan and Consedine, 2014). Our study investigated brain response changes to three emotional stimuli as a result of a special AT practice developed for headache patients. The observed effect differed depending on the diagnosis. Healthy subjects showed greater activation change in the supplementary motor area (SMA) of the left medial frontal gyrus compared with migraineurs to fearful faces, while migraine subjects showed greater activation change in the left insula to happy faces compared with healthy subjects. In addition, decreased activity in the migraine-associated pons region was measured in the migraine subsample. Migraineurs also reported a significant reduction in the number of migraine attacks in response to AT.

### Impact of Autogenic Training Developed for Headache Patients on the Number of Migraine Attacks

Hereby, we provide further evidence of the prophylactic effect of AT already proved by Zsombok et al. (2003) and Juhasz et al. (2007). Regular practice of AT set up specifically for patients with headaches resulted in a decreased number of migraine attacks in female episodic migraineurs. In our study, the number of attacks measured in the month prior to the first fMRI scanning dropped just before the completion of the training. Numerous studies have already reported the positive impact of behavioral treatments on migraine parameters (Janssen and Neutgens, 1986; Lemstra et al., 2002; Meyer et al., 2016). However, the measurement is not easy, since studies are quite heterogeneous with respect to the population (e.g., type of headache, chronic or episodic migraineurs, sample size), the design (e.g., length of the study and the follow-up period, type of control groups, measurement of headache parameters), and the intervention (e.g., type of the intervention, application of therapies alone or in combination with other treatments). Therefore, one has to be cautious in comparing their results with each other or generalizing their achievements.

Minen et al. (2018) assessed factors inhibiting migraineurs from initiating behavior treatments. The main concerns of responders were time limitation, cost, fear of ineffectiveness, and satisfaction with their current treatment. Patients have to accept that the therapy is time-consuming, but the investment returns if the frequency of attacks reduces. Our study confirms that AT can help reduce migraine frequency, possibly due to its impact on brain activation.

### Neural Activation Changes

Very few studies investigated the effect of behavioral therapies in terms of brain activation, and, to our knowledge, none of them analyzed it in relation to emotion processing in migraine. Schlamann et al. (2010) measured the activation patterns during AT in healthy men and women experienced in AT and in non-experienced controls. They detected higher activation in the left prefrontal cortex, as well as in the bilateral post- and precentral cortex of the experienced group compared with the non-experienced group. Furthermore, they found a positive correlation between the number of years of AT and the activation of the insula that is responsible for the integration of sensory and emotional information and influences the affective response (Derbyshire, 2003). Naglatzki et al. (2012) wanted to explore the mechanisms of pain modulation induced by AT. Cerebral activation in the resting state was compared with activation assessed during the painful stimulus. AT and non-AT sessions were performed under the same experimental conditions. Subjects were healthy men and women having practiced AT for years. Several pain modulatory regions showing increased activation during non-AT scanning (e.g., anterior midcingulate cortex, right anterior insular cortex, right thalamus) became less active during the AT session, while the left ventrolateral prefrontal cortex was more active. This can be the manifestation of a coping mechanism for the emotional impact of pain (Salomons et al., 2007). Kobayashi and Koitabashi (2016) investigated the effect of the PMR, a method close to AT, on healthy men. In contrast to the control session (muscles simply tensed and relaxed), the PMR resulted in less areas with increased activation, and some regions, such as the superior frontal gyrus, deactivated which is a sign of exclusion of distractions and focusing on relaxation according to the authors. It is important to note that these studies investigated healthy subjects. However, their findings are in line with our observations in terms of the modulatory effect of AT on regions participating in emotion regulation, pain modulation, and concentration.

Interestingly, in our study, observed activation changes are located on the left side of the brain both in between-group and within-group analysis. Lateralization of pain is supposed to be caused by lateralized brain dysfunction (Afridi et al., 2005) and is also affected by hand dominance (La Pegna et al., 2021). However, we cannot establish any relationship between localization of pain, laterality of activation change, and handedness, because our subjects were all right-handed and have variable headache laterality.

#### Autogenic Training Reduced Pons Activation in Migraineurs

Change in the pons activation may be a characteristic specific to migraineurs. Decreased activity level (see Table 3) is an important result, since pons, especially the dorsal area, is thought to play a role in the development of migraine attacks (Stankewitz et al., 2011) and also showed altered activation during pain-free periods in a study of Wilcox et al. (2016). However, the way this region contributes to the onset of a migraine attack is not clarified yet (Borsook and Burstein, 2012).

Numerous nuclei are located in this region, partly associated with certain migraine symptoms including nausea, dizziness, and hypersensitivity (Borsook and Burstein, 2012). The identified area, namely the parabrachial complex (see Figure 1D), is strongly connected to brainstem regions (rostral ventral medulla, periaqueductal gray) and the amygdala. It plays a crucial role in pain modulation probably *via* its CGRP-expressing neurons working as a general alarm in response to nociceptive signals (Chiang et al., 2019). After completing the 16-week AT course, the activation of this area decreased in migraineurs to fearful faces, but not to happy or sad ones. Behind this activation change, we might suppose a possible contribution of CGRP-neurons within the parabrachial complex. These neurons transmit negative valence, strengthen the original fear memory and become active during the recall of such memories (Campos et al., 2018). The important role of CGRP in migraine has already been proved by the efficacy of medicines such as CGRP-receptor blocker gepants, monoclonal antibodies against CGRP and CGRP receptors (Do et al., 2019). By blocking CGRP signaling, they may alleviate migraine pain or prevent migraine attacks (Edvinsson et al., 2018), and AT could be an adjunctive therapy amplifying their effect. Although the current study could not show an unequivocal association between migraine frequency reduction and diminished pons activity, the characteristics of this brainstem area assume a potential relationship. This should be confirmed by future research.

#### Difference of Supplementary Motor Area and Insula Activation Changes Between Controls and Migraineurs

After AT, we detected a difference in brain activity changes between controls and migraineurs within the area of the left medial frontal gyrus. This region corresponds to the SMA, which is associated with several functions including working memory (Cañas et al., 2018), speech (Alario et al., 2006), motor learning (Hardwick et al., 2013), auditory processing (Lima et al., 2016), but it is best known for motor planning (Makoshi et al., 2011). However, studies support that SMA is an interface between the emotional and motor system, it has a modulatory role in emotional perception (Rodigari and Oliveri, 2014; Vergani et al., 2014) and is also involved in fear conditioning (Fullana et al., 2016). Healthy female subjects of Rodigari and Oliveri (2014) evaluated the valence of emotional pictures while repetitive transcranial magnetic stimulation was applied over their SMA. Perception of negative valence (disgust and fear) was increased, while the perception of positive (erotic) emotional stimuli was decreased after stimulation. In our study, fearful facial expressions triggered the activation change of SMA: activation increase in healthy controls and activation decrease in migraineurs were detected after the completion of AT. Thus, AT might have exerted a different effect on the aversive visual input processing in controls and migraineurs, since the direction of the activation change of SMA is opposite in the groups. The baseline activation level of this area significantly differed between the groups, but the levels approached each other by the end of the AT (see the overlap of 95% confidence intervals of After AT activations in Figure 2A and Supplementary Table 5), which may refer to an optimization mechanism in the detection and processing of threatening stimuli. It might be explained by the presumed role of SMA in cognitive control (Nachev et al., 2008): control subjects may have learned to focus on their inner sensations, while migraineurs may have managed to decrease their excessive attention to aversive stimuli.

The difference between the two groups was also reflected by the activation change of the insula, a crucial hub of affective processing. This region called the “limbic integration cortex” (Borsook et al., 2016) has a role—among others—in interoceptive awareness and emotion processing (Pavuluri and May, 2015). Reaction to affective stimuli is influenced by how the insula interprets incoming sensations (Pavuluri and May, 2015). Alterations have been found in the functioning and connections of the insula (Borsook et al., 2016; Lee et al., 2017) mainly in the processing of negative emotional stimuli and pain (Mathur et al., 2016). In our study, we experienced increased activation to positive stimuli (happy faces) in the left posterior insula of migraineurs (see Figures 1B, 2B). Migraineurs are often characterized by negative thinking that hampers them from developing adaptive coping strategies (Chan and Consedine, 2014). Concerning the insula, there are inconsistent findings in relation to stimulus valence and lateralization. A meta-analysis of Duerden et al. (2013) found the following. The stimulation of the left insula induces parasympathetic responses like a sense of safety and experience of positive affect. However, the lowest activation has been observed in the left posterior insula relative to other insular regions to positive emotional stimuli. Nonetheless, the left hemisphere was activated to a greater extent than the right one. In females, the dominance of the left side of the posterior insula has been observed in all types of emotional stimuli, while males show higher activation on the right side. Insula has a posterior-to-anterior direction of stimulus processing: sensory-motor information is received and interpreted by the posterior insula as bodily experiences, then transferred to the anterior part where awareness of feelings and subjective experiences are developed (Pavuluri and May, 2015). This applies to nociceptive inputs as well (Borsook et al., 2016). In our study, the activation level of the left posterior insula increased in migraineurs, while decreased in the control group. The higher activation level of this region observed in migraineurs as compared with controls after AT may indicate a change resulting in the easier detection of pleasant emotional stimuli among migraineurs. Controls showed a significantly decreased activation, but no deactivation compared with before AT level. This is in contrast to the results of Schlamann et al. (2010) who found a correlation between the experience in AT and the insular cortex activation. All our subjects, both controls and migraineurs, had the same level of experience, and no correlation could be revealed between the AT frequency and the insula activation change. However, two studies using acupuncture, a non-pharmacological anti-migraine treatment which cannot be considered as evidence-based yet (Campbell et al., 2000) reported significant pain reduction followed by increased activation of the left posterior insula in migraineurs compared to healthy controls (Yang et al., 2012; Li et al., 2017). Since insula is involved in the affective aspects of pain, improvement in headache parameters and increased reaction to positive emotional stimuli may be associated.

## Limitations

Due to the fact that participation in the AT course could be offered to a limited number of subjects, some of whom did not follow the course regularly and given the unbalanced gender ratio in the migraine group, the final sample was small-sized and consisted of solely female participants. The small size could be the reason why no correlation between pons activation decrease and change in the number of attacks was found. However, sex homogeneity gave us the possibility to examine a population particularly impacted by migraine. Since most of the exercises were performed individually, only learning sessions required personal attendance, the AT frequency was measured by self-reported forms. Similarly, retrospective reports were used to assess other clinical parameters including the number of migraine attacks. This may bias the results, though retrospective reports have been shown to be reliable in the assessment of headache frequency (Niere and Jerak, 2004). AT frequency defined as an inclusion criterion was not unified, but a minimum level (once a week) was required. Therefore, certain participants practiced more than others. Also, it is important to mention that we did not have a migraineur group performing the fMRI task a second time without practicing AT. The involvement of such a group could have improved the power of our observations. Another limitation of the study is that the headache frequency of migraine subjects was not followed up for a longer period after the AT course. Concerning the fMRI scans, they were smoothed with a kernel of 8 mm which is larger than recently used ones and not ideal for smaller brain areas such as the pons and hypothalamus. However, kernel size is important for voxel-wise whole-brain analyses, while it has little effect on ROI analysis (Huettel et al., 2014). Finally, although the identification accuracy of dorsal pons area was limited due to its small size and low resolution of fMRI, both the Harvard Ascending Arousal Network Atlas and characteristics of parabrachial complex support its correctness.

## Conclusion

Our pilot study is the first one investigating the effect of AT on brain activation of migraineurs compared with healthy controls. After using an intervention tailored to the needs of headache patients, a decreased number of migraine attacks were reported by the patients further supporting the effectiveness of AT in migraine therapy. The effect of AT was also demonstrated *via* the detection of brain activation changes to fearful and happy (but not sad) emotional stimuli in both groups. Among migraineurs, a reduced activation was found in the parabrachial complex within the migraine-associated region of dorsal pons. Concerning group differences, the activation of the emotional processing- and cognitive control-related SMA increased in healthy controls compared with migraineurs. Interestingly, the activation level of this region was more similar between the two groups after the intervention possibly showing an optimizing effect of AT on the processing of threatening fearful stimuli. Regarding the increase of left posterior insula activation to happy faces in the migraine group, this change might be due to a greater openness to positive emotional stimuli. Our results suggest that AT may be an effective adjunctive behavioral therapy in migraine contributing to decreased attack frequency and changes in activation of brain regions involved in migraine, pain modulation, and emotional processing.

## Data Availability Statement

The datasets presented in this article are not readily available because they contain information that could compromise the privacy of research participants. Requests to access the datasets should be directed to GJ, juhasz.gabriella@pharma.semmelweis-univ.hu.

## Ethics Statement

The study as reviewed and approved by the Scientific and Research Ethics Committee of the Medical Research Council of Hungary (23609-1/2011-EKU and 23421-1/2015/EKU) and was carried out in accordance with the Declaration of Helsinki. Written informed consent forms were obtained from all subjects.

## Author Contributions

DD contributed to the formal analysis, methodology, visualization, writing original draft, reviewing, and editing. ES contributed to the data curation, formal analysis, investigation, methodology, writing, reviewing, and editing. DB, KG, NK, and DP contributed to the data curation, investigation, writing, reviewing, and editing. TZ, LRK, and GK contributed to the investigation, writing, reviewing, and editing. GJ contributed to the conceptualization, formal analysis, funding acquisition, investigation, methodology, project administration, resources, supervision, validation, writing, reviewing, and editing. All authors contributed to the article and approved the submitted version.

## Conflict of Interest

The authors declare that the research was conducted in the absence of any commercial or financial relationships that could be construed as a potential conflict of interest.

## Publisher’s Note

All claims expressed in this article are solely those of the authors and do not necessarily represent those of their affiliated organizations, or those of the publisher, the editors and the reviewers. Any product that may be evaluated in this article, or claim that may be made by its manufacturer, is not guaranteed or endorsed by the publisher.

## Footnotes

1
https://www.fil.ion.ucl.ac.uk/spm/software/spm12/

2
https://www.nitrc.org/projects/artifact_detect

3
https://www.mccauslandcenter.sc.edu/mricrogl/
