# Supplementary material for: Regular Practice of Autogenic Training Reduces Migraine Frequency and Is Associated With Brain Activity Changes in Response to Fearful Visual Stimuli
Source: Front Behav Neurosci. 2022 Jan 21;15:780081. doi: 10.3389/fnbeh.2021.780081 (PMC8814632; doi:10.3389/fnbeh.2021.780081)
Supplement: Supplementary file 1 [file Table_1.docx]

**Supplementary Material**

**Supplementary Methods.** Description of headache-specific autogenic training.

In order to be more beneficial for headache patients, the Schultz-type autogenic training has been completed with extra exercises that help patients experience serenity, be more aware of their body and be able to relieve muscle tension of the back-neck-shoulder regions. The first session was dedicated to psychoeducation: the therapist talked about the physiological and psychological effects of AT, and the optimal physical setting for relaxation. During the next thirteen sessions, the following classical Schultz-type exercises were taught to patients: heaviness experience (muscular relaxation), experience of warmth (vascular dilation), regulation of the heart, regulation of breathing, regulation of visceral organs, and regulation of the head (for details, see Linden, 1993). Extra sessions focused on headache-specific exercises: (1) relaxing and warming up back, neck and shoulders musculature can prevent the development of headache pain; (2) experiencing serenity ‘Serenity pervades my whole body.’ has an anxiolytic and energizing effect on patients; (3) imagination of shrinkage and of enlargement of the body ‘My body is shrinking…My body is small. … My body is growing. … My body is large.’ can enhance body awareness. During the closing session, patients shared their overall experiences and could ask questions. Throughout the entire training course, patients were encouraged to share and discuss their experiences, sensations and thoughts spontaneously arising during their practices.

**Supplementary Table S1.** Number of migraine headaches in the last month before the first and the second fMRI scanning per migraine subject.

| **Subject** | **Number of migraine headaches** | | |
| --- | --- | --- | --- |
|  | **Before AT** | **After AT** | **After AT vs. Before AT** |
| 1 | 10 | 7 | **-3** |
| 2 | 3 | 2 | **-1** |
| 3 | 4.5 | 4 | **-0.5** |
| 4 | 5 | 3 | **-2** |
| 5 | 8 | 1 | **-7** |
| 6 | 10 | 8 | **-2** |
| 7 | 11 | 4 | **-7** |
| 8 | 4 | 2 | **-2** |
| 9 | 3 | 2 | **-1** |
| 10 | 4 | 5 | **1** |
| 11 | 5 | 0 | **-5** |

**Supplementary Table S2.** Accuracy level and reaction time in sex identification fMRI task.

| **(A)** | **Before AT** | | **After AT** | |
| --- | --- | --- | --- | --- |
| **Mean accuracy across all conditions** | | | | |
|  | 98.75% (SD = 2.39) | | 98.95% (SD = 3.30) | |
| **Difference in mean accuracy across all conditions between controls and migraineurs** | | | | |
|  | *U* | *p* | *U* | *p* |
|  | 73.5 | 0.622 | 73.5 | 0.567 |
| **Difference in reaction times between controls and migraineurs** | | | |  |
|  | *t* | *p* | *t* | *p* |
| neutral | -1.259 | 0.22 | -0.793 | 0.44 |
| fear | -1.918 | 0.067 | -0.643 | 0.526 |
| happy | -1.155 | 0.259 | -0.332 | 0.744 |
|  | *U* | *p* |  |  |
| sad | 71 | 0.551 | -0.212 | 0.834 |

| **(B)** | **Controls** | | **Migraineurs** | |
| --- | --- | --- | --- | --- |
| **Difference in mean accuracy across all conditions: before AT vs. after AT** | | | | |
|  | *Z* | *p* | *Z* | *p* |
|  | -1.051 | 0.293 | -0.71 | 0.478 |
| **Difference in reaction times: before AT vs. after AT** | | |  |  |
|  | *t* | *p* | *t* | *p* |
| neutral | -0.926 | 0.37 | -0.076 | 0.941 |
| fear | -0.798 | 0.438 | 1.59 | 0.143 |
| happy | -1.743 | 0.103 | -0.137 | 0.894 |
|  |  |  | *Z* | *p* |
| sad | -0.442 | 0.665 | -0.089 | 0.929 |

*Note.* **(A)** Between-groups analyses were carried out using independent samples t-test and Mann-Whitney U test, while **(B)** within-groups analyses were performed using Wilcoxon signed-rank test and paired samples t-test. SD = standard deviation.

**Supplementary Table S3.** Significant main effects of emotional faces task per emotion before and after AT.

|  | **Cluster size** | **p_FWE_** | **Region** | **Peak coordinates** | | | **Peak T-value** | |
| --- | --- | --- | --- | --- | --- | --- | --- | --- |
|  |  |  |  | x | y | z | |  |
| **Before AT** |  |  |  |  |  |  | |  |
| ***Controls & Migraineurs*** | | |  |  |  |  | |  |
| Neutral-Rest | 263 | 0.000 | L Postcentral Gyrus | -51 | -28 | 56 | | 6.74 |
|  | 103 | 0.033 | R Frontal Operculum | 51 | 11 | 2 | | 5.52 |
| Fearful-Neutral | 88 | 0.043 | R Precentral Gyrus | 51 | 2 | 32 | | 5.17 |
| ***Controls*** |  |  |  |  |  |  | |  |
| Neutral-Rest | 1689 | 0.000 | R Fusiform Gyrus | 39 | -52 | -22 | | 11.65 |
|  | 90 | 0.028 | L Postcentral Gyrus | -51 | -31 | 56 | | 5.68 |
| Sad-Neutral | 1470 | 0.000 | L Cuneus | -9 | -94 | 14 | | 9.24 |
|  | 82 | 0.035 | R Inferior Temporal Gyrus | 42 | -10 | -28 | | 8.84 |
| ***Migraineurs*** |  |  |  |  |  |  | |  |
| Neutral-Rest | 767 | 0.000 | R Cerebelum VI | 9 | -82 | -19 | | 7.83 |
| Fearful-Neutral | 59 | 0.010 | L Hippocampus | -21 | -7 | -22 | | 12.63 |
| **After AT** |  |  |  |  |  |  | |  |
| ***Controls & Migraineurs*** | | |  |  |  |  | |  |
| Neutral-Rest | 459 | 0.000 | L Precentral Gyrus | -39 | -19 | 59 | | 7.19 |
|  | 218 | 0.000 | R Supplementary Motor Area | 3 | 8 | 59 | | 5.53 |
| Happy-Neutral | 108 | 0.002 | R Paracentral Lobule | 12 | -22 | 71 | | 6.19 |
| ***Controls*** |  |  |  |  |  |  | |  |
| Neutral-Rest | 221 | 0.000 | L Postcentral Gyrus | -54 | -22 | 50 | | 7.21 |
| Fearful-Neutral | 954 | 0.000 | R Inferior Temporal Gyrus | 48 | -49 | -16 | | 9.11 |
| Sad-Neutral | 357 | 0.000 | R Inferior Temporal Gyrus | 45 | -52 | -16 | | 6.45 |
| ***Migraineurs*** |  |  |  |  |  |  | |  |
| Neutral-Rest | 131 | 0.000 | L Hippocampus | -15 | -4 | -22 | | 10.17 |
|  | 1290 | 0.000 | R Cerebelum VI | 21 | -64 | -22 | | 10.07 |
|  | 47 | 0.039 | L Calcarine | -15 | -97 | -4 | | 5.24 |
| Happy-Neutral | 146 | 0.000 | R Postcentral Gyrus | 60 | -4 | 20 | | 10.49 |
|  | 61 | 0.004 | R Putamen | 33 | -1 | 5 | | 10.38 |
|  | 40 | 0.032 | R Precentral Gyrus | 15 | -28 | 77 | | 8.14 |
|  | 37 | 0.044 | L Middle Temporal Gyrus | -57 | -67 | -4 | | 7.43 |
|  | 83 | 0.001 | R Cerebelum VI | 36 | -46 | -25 | | 7.28 |
| Sad-Neutral | 182 | 0.000 | R Inferior Temporal Gyrus | 48 | -40 | -16 | | 7.50 |
|  | 38 | 0.043 | L Superior Parietal Gyrus | -15 | -67 | 53 | | 7.06 |

*Note.* Significant clusters are shown at cluster-level p_FWE_ < 0.05 in MNI coordinates. (R = right, L = left) Neutral-rest contrast represents brain areas activated by looking at neutral faces compared to fixation cross. Fearful-neutral, Sad-neutral, and Happy-neutral contrasts represent brain areas activated by the relevant emotion compared to neutral faces.


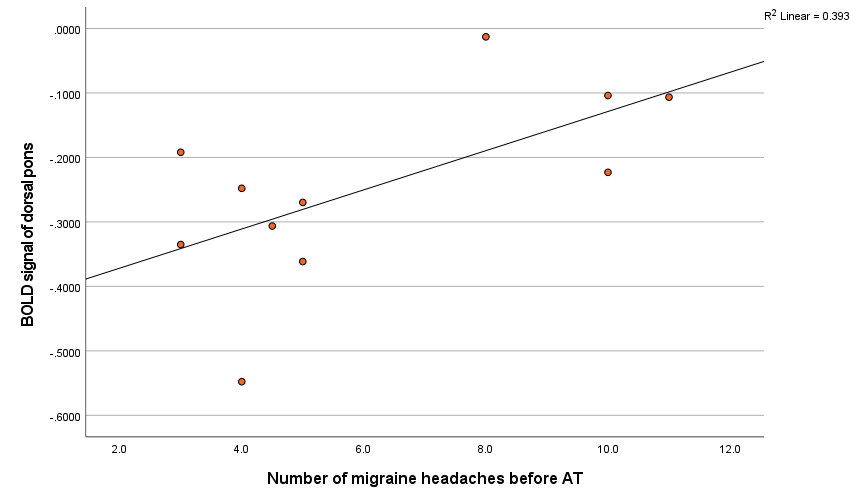


**Supplementary Figure S1.** Trend significance of negative correlation between the number of migraine headaches in the last month before the first fMRI scanning and the deactivation of dorsal pons to fearful emotional stimuli in the migraine subsample (Spearman’ rho = 0.541, p = 0.085).

**Supplementary Table S4.** Significant intragroup activation change after AT vs. before AT within the brain areas showing different activation change between groups (Left medial frontal gyrus: x = 0, y = -16, z = 65; Left insula: x = -45, y = -13, z = 5).

|  | **Test statistics** | **p value** |
| --- | --- | --- |
| **Left medial frontal gyrus (SMA)** |  |  |
| Controls | 12 | 0.006 |
| Migraineurs | 61 | 0.013 |
| **Left insula** |  |  |
| Controls | 100 | 0.023 |
| Migraineurs | 3 | 0.008 |

*Note.* Wilcoxon signed-rank test.

**Supplementary Table S5**. Significantly different activation level of left medial frontal gyrus (SMA, x = 0, y = -16, z = 65) and of left insula (x = -45, y = -13, z = 5) between controls and migraineurs before and after AT.

|  | **Test statistics** | **p value** |
| --- | --- | --- |
| **Before AT** |  |  |
| Left medial frontal gyrus (SMA) | 23 | 0.002 |
| Left insula | 21 | 0.001 |
| **After AT** |  |  |
| Left medial frontal gyrus (SMA) | 42 | 0.036 |
| Left insula | 36 | 0.016 |

*Note.* Mann-Whitney U test
